# Expression of Endometrial Receptivity Markers throughout the Menstrual Cycle in Women with and without Uterine Adenomyosis

**DOI:** 10.3390/jcm13175016

**Published:** 2024-08-24

**Authors:** Christina Anna Stratopoulou, Ines El Grari, Alessandra Camboni, Jacques Donnez, Marie-Madeleine Dolmans

**Affiliations:** 1 Gynecology Research Unit, Institute of Experimental and Clinical Research, Université Catholique de Louvain, 1200 Brussels, Belgium; christina.stratopoulou@uclouvain.be (C.A.S.);; 2 Anatomopathology Department, Cliniques Universitaires Saint-Luc, 1200 Brussels, Belgium; 3 Société de Recherche Pour l’Infertilité, 1150 Brussels, Belgium; 4 Université Catholique de Louvain, 1200 Brussels, Belgium; 5 Gynecology Department, Cliniques Universitaires Saint-Luc, 1200 Brussels, Belgium

**Keywords:** adenomyosis, endometrial receptivity, decidualization, progesterone resistance, window of implantation, infertility

## Abstract

**Background/Objectives**: While it is known that adenomyosis is associated with poor reproductive outcomes, the underlying mechanisms are unclear, and to date, there is no standard treatment protocol for these patients. Endometrium from adenomyosis patients is characterized by several abnormalities, potentially resulting in impaired receptivity and subsequent implantation failure. **Methods**: Endometrial biopsies were collected from 26 women with adenomyosis and 26 control subjects. Immunohistochemistry was performed to evaluate the expression of markers of endometrial receptivity, namely the progesterone receptor (PR), glycodelin, leukemia inhibitory factor (LIF), homeobox A10 (HOXA10), integrin beta chain beta 3 (integrin β3) and osteopontin. Scanning electron microscopy was used to observe pinopodes on the surface of mid-secretory endometrial epithelium. **Results**: PR, LIF and osteopontin expression were all found to be weaker in secretory-phase stroma from adenomyosis patients than in healthy controls. HOXA10 expression was decreased in adenomyosis during the secretory phase, and also the proliferative phase, where it reached statistical significance in both epithelial and stromal compartments. Glycodelin and integrin β3 levels did not differ between diseased and healthy tissues in any of the cycle phases. Pinopodes were fewer and at later developmental stages in adenomyosis compared to those on the surface of healthy endometrium from the same time period of the menstrual cycle. **Conclusions**: Endometrium from adenomyosis patients is characterized by abnormal expression of various receptivity markers. The stromal compartment appears to be affected most, showing reduced expression of PR, LIF and osteopontin in the secretory phase and lower levels of HOXA10 during both proliferative and secretory phases. Decreased receptivity due to impaired stromal decidualization may contribute to poor reproductive outcomes in adenomyosis patients.

## 1. Introduction

Embryo implantation is a complex and strictly regulated process depending on both the blastocyst and maternal endometrium. In humans, endometrium has the unique ability to self-prepare for blastocyst implantation every month, irrespective of the presence of a fertilized oocyte, during a period known as the window of implantation (WOI, days 7–9 within the secretory phase) [1]. During the WOI, fundamental changes occur in both main types of endometrial cells, namely epithelial and stromal cells [1,2]. Epithelial glands become actively secretory at this stage, releasing glycoproteins to facilitate blastocyst adherence and provide the growing embryo with nutrients throughout the first trimester of pregnancy [3]. Stromal cells, on the other hand, respond to progesterone by undergoing a process called decidualization, which involves their morphological and functional transformation into secretory cells, ultimately promoting trophoblast invasion and development of the placenta [2,4]. Disruption of these tightly regulated processes, as in the case of progesterone resistance, may lead to implantation failure and subsequent subfertility.

Despite being initially considered a disease of older multiparous women, it is now clear that adenomyosis also affects younger women, compromising their reproductive potential among other things. Meta-analyses increasingly confirm a link between adenomyosis and poor reproductive outcomes in the in vitro fertilization (IVF), including lower rates of embryo implantation, fewer clinical pregnancies per cycle, higher miscarriage rates and an increased risk of pregnancy complications [5,6,7,8]. Mechanisms underlying adenomyosis-related implantation failure are not yet fully understood but are considered to be due to an altered endometrial microenvironment. While many studies have attempted to investigate the impact of adenomyosis on fertility outcomes, none have yielded robust conclusions or led to the development of a standard medical protocol to treat the condition [9,10]. It is therefore vital to better understand and diagnose the fundamental causes of infertility in these patients.

Certain proteins can be evaluated to determine the capacity of the endometrium to respond to the hormonal milieu and self-prepare for implantation, often referred to as receptivity markers. We therefore conducted a retrospective study to investigate the expression of some of the most important fertility markers, namely the progesterone receptor (PR), glycodelin, leukemia inhibitory factor (LIF), homeobox A10 (HOXA10), integrin beta chain beta 3 (integrin β3), osteopontin and pinopodes. The aim of the study was to compare the expression of these receptivity markers in endometrium from both adenomyosis patients and healthy women according to the different phases of the menstrual cycle. 

## 2. Materials and Methods

### 2.1. Study Participants and Sample Collection

Use of human tissues for this study was undertaken according to the ethical principles of the Declaration of Helsinki and approved by the Ethics Committee of Cliniques Universitaires Saint-Luc (CUSL) and the Université Catholique de Louvain on 31 August 2020 and amended on 23 July 2023 (reference 2020/14AOU/410).

Twenty-six adenomyosis patients and 26 control subjects presenting to the gynecology department of CUSL were included in the study. Residual endometrial specimens from standard clinical procedures (hysterectomy, diagnostic curettage) were collected and fixed in 4% formaldehyde and embedded in paraffin for further use.

Adenomyosis was diagnosed on the basis of imaging (magnetic resonance imaging or transvaginal ultrasound) and further confirmed by histology on hysterectomy specimens, when available. All patients presented with diffuse adenomyosis involving myometrial heterogeneity and junctional zone thickening. The control group included women with no sign of adenomyosis or endometriosis, who were either undergoing endometrial biopsy for diagnostic purposes or hysterectomy for conditions unrelated to the endometrium, such as intramural myomas or cervical carcinoma. All the women were premenopausal and none of them had been under the influence of (hormonal) contraception or selective sex steroid modulators for at least 2 months prior to intervention. Clinicopathological characteristics of the recruited patients are listed in Table 1. Mean age was higher in the adenomyosis group compared to the healthy group (*p* = 0.0286). Concerning the parity, this parameter was also statistically different between the groups (*p* = 0.03), but in the majority of adenomyosis patients, pregnancies occurred before the age of 30, prior to disease development. Last, the rates of menorrhagia (*p* = 0.0486) and dysmenorrhea (*p* = 0.0481) were found to be significantly higher in adenomyosis patients compared to healthy women. 

### 2.2. Immunohistochemistry

Five-μm sections were deparaffinized and sequentially rehydrated in serial baths containing Histosafe, toluene, isopropanol and demineralized water. Endogenous peroxidases were blocked using hydrogen peroxide solution, followed by antigen retrieval in citrate buffer. Nonspecific binding sites were blocked in a tris-buffered saline solution containing 1% fetal bovine serum and 0.1% bovine serum albumin. The slides were incubated overnight at 4 °C with primary antibodies targeting PR (A0098, Agilent, CA, USA), glycodelin (PA5-54152, Thermo Fisher Scientific, MA, USA), LIF (PA5-79600, Thermo Fisher), HOXA10 (BS-2502R, Thermo Fisher), integrin β3 (ZRB1515, Merck, Darmstadt, Germany) and osteopontin (ab69498, Abcam, Cambridge, UK). Appropriate tissue specimens were used as positive controls and representative pictures can be found in Supplementary Figure S1. For negative controls, endometrial sections were incubated with the universal negative control for rabbit (IR600, Agilent, CA, USA) or mouse (IR750) instead of primary antibodies. The slides were then rinsed and incubated with secondary anti-rabbit or anti-mouse antibodies for 60 min at room temperature. Diaminobenzidine (DAB) was used for brown colorimetric product formation visible under a brightfield microscope. Counterstaining was done using Mayer’s hematoxylin before dehydration and mounting. Additional data on the solutions and dilutions used are reported in Supplementary Table S1. 

### 2.3. Image Acquisition and Staining Quantification

Tissue sections were digitized using the Pannoramic SCAN II (Sysmex, Terhulpen, Belgium) at 20× magnification. For glycodelin immunohistochemistry, rates of positive glands per total glands were manually counted in 10 different tissue fields. Analyses for the progesterone receptor, LIF, HOXA10, integrin β3 and osteopontin were conducted using the Visiopharm version 2022.01 (Hamamatsu, Mont-Saint-Guibert, Belgium) to calculate the staining index, taking into account both staining intensity and percentage of the labeled area [11]. The epithelial and stromal compartments were manually surrounded and separately analyzed. 

### 2.4. Statistical Analysis

All statistical analyses were performed using GraphPad Prism, version 9.5.1; GraphPad Software, Boston, MA, USA. One-way ANOVA or the Kruskal-Wallis test followed by multiple comparisons were used to compare normally and non-normally distributed data respectively. Multiple regression analysis using the least squares approach was conducted to confirm variables affecting the expression of different markers (Supplementary Table S2). *p*-values below 0.05 were considered significant.

### 2.5. Scanning Electron Microscopy (SEM)

Representative endometrial samples (one per group) derived from the mid-secretory phase were used for SEM imaging. To this end, tissues were quickly minced into ±1 mm^3^ fragments and submerged in 2% paraformaldehyde/2.5% glutaraldehyde in phosphate buffer (0.1 M; pH 7.4) overnight at 4 °C. The fragments were then dehydrated in a graded series of ethanol, dried inside a vacuum chamber, and gold-plated. Finally, they were inspected under the JEOL JSM-IT200 microscope. A total of 10 fields per sample were examined.

## 3. Results

### 3.1. Progesterone Receptor

**Figure 1:**
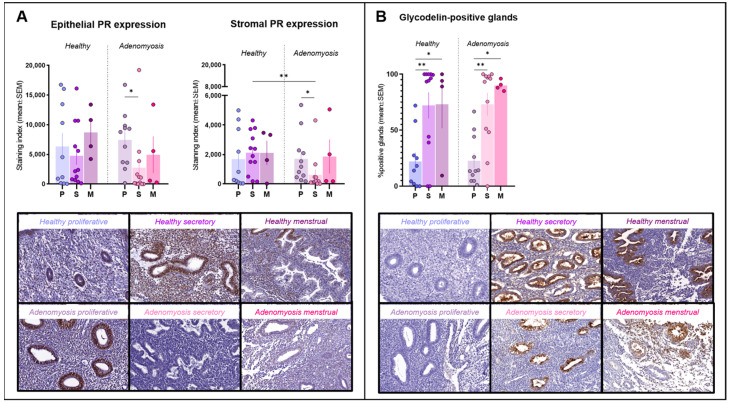
Progesterone receptor (PR) and glycodelin immunostaining. (**A**) Epithelial and stromal PR expression in healthy endometrium and adenomyosis according to the different phases of the menstrual cycle (epithelium: * *p* = 0.0185; stroma: ** *p* = 0.004; * *p* = 0.0102,). (**B**) Percentages of glycodelin-positive glands in healthy endometrium and adenomyosis according to the different phases of the menstrual cycle (** *p* = 0.0024; 0.005, * *p* = 0.0452; 0.0357).

PR immunostaining was observed inside nuclei from both epithelial and stromal cells (Figure 1A). No significant differences in expression were detected between adenomyosis and healthy endometrium in the epithelial compartment. In the stroma, however, PR expression was significantly lower during the secretory phase in adenomyotic tissues than in healthy tissues from the same phase of the cycle (*p* = 0.004). We also observed a significant drop in both epithelial (*p* = 0.0185) and stromal (*p* = 0.0102) immunostaining for PR in the secretory phase compared to the proliferative phase in the case of adenomyosis. 

### 3.2. Glycodelin

Glycodelin immunostaining was detected only in glands, where it appeared both in cells and within the lumen (Figure 1B). Percentages of positive glands increased significantly during the secretory phase compared to the proliferative phase in both healthy (*p* = 0.0014) and adenomyotic (*p* = 0.005) endometrium. Similarly, rates of positive staining were found to be higher in menstrual than in proliferative endometrium in healthy (*p* = 0.0452) and adenomyotic (*p* = 0.0357) tissues. No differences were observed between healthy endometrium and adenomyosis during any of the phases of the menstrual cycle.

### 3.3. Leukemia Inhibitory Factor

**Figure 2:**
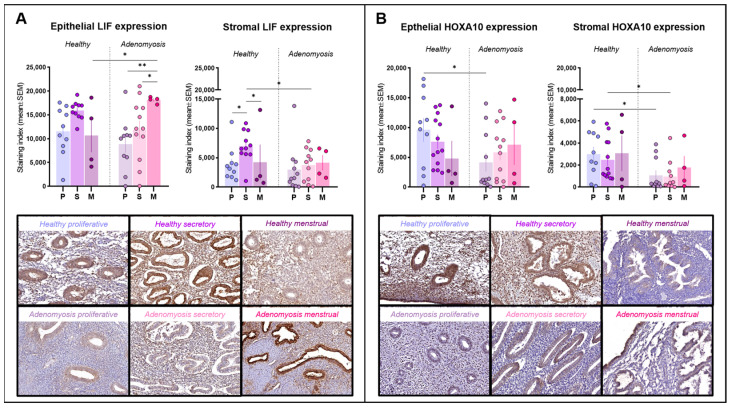
Leukemia inhibitory factor (LIF) and homeobox A10 (HOXA10) immunostaining. (**A**) Epithelial and stromal LIF expression in healthy endometrium and adenomyosis according to the different phases of the menstrual cycle (epithelium: * *p* = 0.0446; 0.0393, ** *p* = 0.0034; stroma: * *p* = 0.0381; 0.0499; 0.0253). (**B**) Epithelial and stromal HOXA10 expression in healthy endometrium and adenomyosis according to the different phases of the menstrual cycle (epithelium: * *p* = 0.0198; stroma: * *p* = 0.0205; 0.0198).

LIF immunoreactivity was detected primarily within the cytoplasm of epithelial and stromal cells (Figure 2A). LIF expression was found to be very high in menstrual epithelium from adenomyosis patients, significantly higher than that during the proliferative (*p* = 0.0034) or secretory (*p* = 0.0393) phases in the adenomyosis group. Menstrual-phase glands from adenomyosis patients also showed a significantly higher expression of LIF than those from healthy tissues (*p* = 0.0446). In the stroma, LIF immunostaining was stronger in secretory-phase endometrium than in proliferative (*p* = 0.0381) or menstrual (*p* = 0.0499) endometrium in healthy controls, but this trend was not maintained in the case of adenomyosis. During the secretory phase, stromal expression of LIF was significantly lower in adenomyotic tissues than in healthy endometrium (*p* = 0.0253). 

### 3.4. Homeobox A10

HOXA10 was detected inside cell nuclei in both epithelial and stromal cells (Figure 2B). In the epithelium, HOXA10 expression was intense in healthy endometrium from the proliferative phase, significantly stronger than that from adenomyosis patients in the same cycle phase (*p* = 0.0198). In the stromal compartment, HOXA10 expression remained low during both the proliferative and secretory phases in adenomyosis and was significantly different from the corresponding phases in healthy controls (*p* = 0.0205; 0.0198).

### 3.5. Integrin Beta Chain Beta 3 

**Figure 3:**
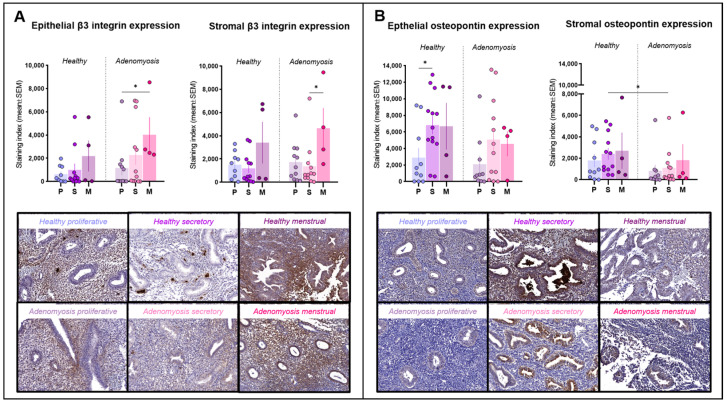
Integrin β3 and osteopontin immunostaining. (**A**) Epithelial and stromal integrin β3 expression in healthy endometrium and adenomyosis according to the different phases of the menstrual cycle (epithelium: * *p* = 0.03; stroma: * *p* = 0.0292). (**B**) Epithelial and stromal osteopontin expression in healthy endometrium and adenomyosis according to the different phases of the menstrual cycle (epithelium: * *p* = 0.0275; stroma: * *p* = 0.0301).

Positivity for integrin β3 was detected mainly in the cytoplasm of epithelial and stromal cells and inside blood vessels (Figure 3A). Its expression did not differ significantly between adenomyotic tissues and healthy endometrium in either the epithelial or stromal compartments. In adenomyosis, menstrual epithelium expressed more integrin β3 than proliferative epithelium (*p* = 0.03). Menstrual stroma also showed high levels of integrin β3, exhibiting a statistically significant difference from the secretory phase (*p* = 0.0292).

### 3.6. Osteopontin

Osteopontin immunolabeling was positive in both the glandular and stromal compartments and localized inside cells in the cytoplasm or secreted (Figure 3B). In healthy endometrium, epithelial osteopontin expression was higher in the secretory phase than in the proliferative phase in the same group (*p* = 0.0275). In the stroma, its secretory-phase expression was significantly weaker in adenomyotic tissues compared to healthy tissues (*p* = 0.0301).

### 3.7. Pinopodes

**Figure 4:**
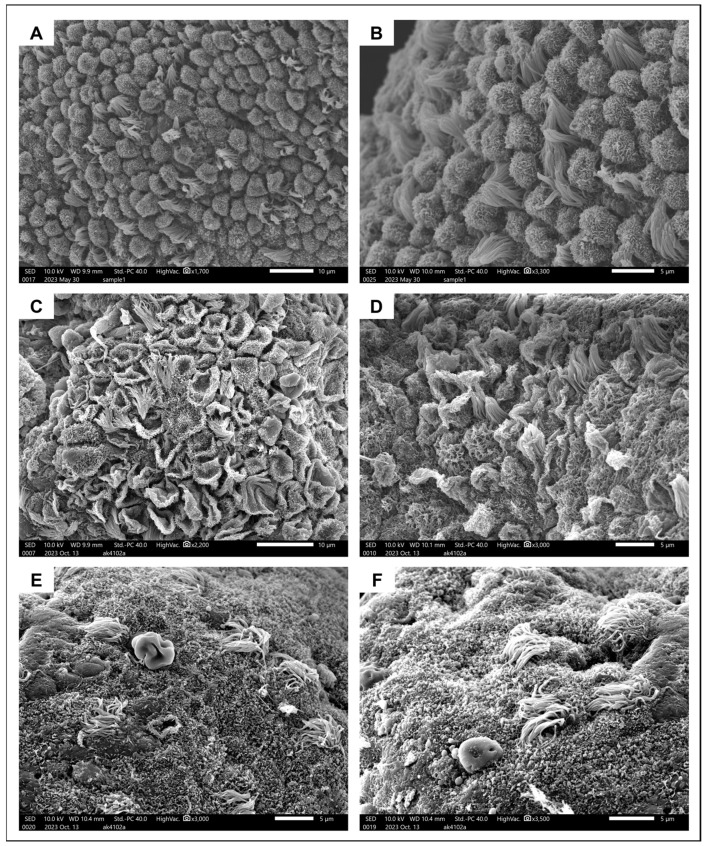
Ultrastructural appearance of pinopodes. (**A**,**B**) Surface epithelium from the healthy endometrium covered in fully formed developing pinopodes interposed with ciliated cells. (**C**,**D**) Regressing pinopodes and several ciliated cells on endometrial surface epithelium from an adenomyosis patient. (**E**,**F**). Different areas of endometrium from the same adenomyosis patient, where pinopodes are now completely absent and very few ciliated cells remain.

SEM revealed the presence of 5–10-μm-long pinopodes in mid-secretory epithelium from a healthy 31-year-old subject (Figure 4A,B). They were spherical in shape and their surface was covered in microvilli, consistent with their later stages of maturation. Ciliated cells were interposed at a ratio of approximately 1:5. A very different image was obtained when examining the endometrium from a 40-year-old adenomyosis patient in the mid-secretory phase of the menstrual cycle. Some areas of the surface epithelium were covered in regressing pinopodes (popped balloon-like appearance) with barely any ciliated cells in between (Figure 4C,D). In other areas, pinopodes were completely absent, and only a small number of ciliated cells were dispersed around the epithelial surface (Figure 4E,F).

## 4. Discussion

Adenomyosis has frequently been associated with poor reproductive outcomes, the exact causes of which vary and have not yet been fully elucidated [9]. Our study has identified several abnormalities in the expression of endometrial receptivity markers, suggesting impaired receptivity as a mechanism contributing to subfertility in affected women. Progesterone is considered the ‘pregnancy hormone’ thanks to its capacity to modulate the expression of numerous genes in favor of embryo implantation, while inadequate progesterone responsiveness leads to uterine-factor infertility [12,13]. Similar to previous findings on adenomyosis, we once again observed a decrease in PR immunoreactivity in endometrium from adenomyosis patients compared to healthy subjects, corroborating the theory of progesterone resistance as a pathogenic feature of this condition [14,15]. Unfortunately, understanding the exact interaction between progesterone signaling and endometrial receptivity in adenomyosis remains challenging, primarily due to a lack of reliable models to mimic this condition. Preliminary data from both in vitro and in vivo studies suggest reduced progesterone responsiveness in adenomyotic endometrium, accompanied by impaired decidualization and fewer offspring in mice with the condition [16,17]. In consistency with these findings, the latest data from our laboratory using 3D endometrial assembloid cultures demonstrate the inability of this tissue to respond to progesterone treatment and acquire characteristics of receptive endometrium. Overall, the results of the present study support a role for progesterone resistance in impaired endometrial receptivity and subsequent subfertility in adenomyosis patients, which warrants further investigation.

Glycodelin is a glycoprotein secreted by endometrial glands and involved in the regulation of cell proliferation, differentiation, adhesion and motility [18]. It is considered a precondition to establishing a successful pregnancy because of the roles it plays in immunosuppression, fertilization and implantation. These roles are further reinforced by the spatiotemporal expression of the protein, showing a radical upturn when transitioning from the proliferative to the secretory phase. This trend was seen in both healthy and adenomyotic endometrium, suggesting that the mechanisms responsible for glycodelin secretion are not compromised in these patients. Nevertheless, glycodelin actually has four distinct glycoforms, so the expression patterns for each of them need to be investigated in greater depth. 

LIF is another protein regulated by estrogen/progesterone signaling and is known to be critical to endometrial receptivity, while its absence causes implantation failure in mice [19]. LIF expression is generally higher during the WOI in fertile women as opposed to patients experiencing infertility [12,20]. Indeed, we observed an increase in LIF immunostaining in healthy subjects progressing from the proliferative to the secretory phase, but this was not witnessed in adenomyosis, where the stromal LIF expression was significantly weaker than that in healthy endometrium. As LIF has a multifaceted role in human reproduction ranging from promoting stromal decidualization to facilitating blastocyst implantation and subsequent development, its dysregulation may compromise any of these processes and hence drastically affect reproductive outcomes in adenomyosis patients. 

The importance of HOXA10 to reproduction is undeniable and ranges from uterine development during embryonic stages to the establishment of endometrial receptivity and embryo implantation [21]. HOXA10 is a transcription factor mediating the expression of other implantation markers, including a number of integrins, while pinopode development is also dependent on its action [22]. In this study, differential HOXA10 expression was observed between adenomyosis and control patients in both epithelial and stromal tissues. Indeed, proliferative-phase endometrium from adenomyosis patients showed decreased positivity in both epithelium and stroma, and the stromal expression also declined during the secretory phase. Moreover, the regression analysis revealed the presence of adenomyosis to be the only predictor of HOXA10 expression, significantly affecting expression levels within the stroma (Supplementary Table S2). HOXA10 expression is known to be dependent on both the local hormone milieu and pathological factors. In fact, a direct link was reported between myoma-derived transforming growth factor beta-3 and HOXA10 downregulation [23]. It is therefore likely that a similar mechanism exists in adenomyotic lesions, eventually inhibiting the expression of this factor. Given the multifaceted role of this protein in endometrial physiology and receptivity establishment, our findings indicate that its dysregulation is a determining factor in adenomyosis-associated impaired endometrial receptivity. 

Integrins are known to participate in embryo adherence to the endometrium, especially the integrin β3 subunit, which is often used to predict defects in progesterone action and subsequent receptivity [1,24]. Encoded by the ITGB3 gene, integrin β3 is an adhesion molecule key to blastocyst attachment and implantation, primarily via its heterodimer integrin alpha V beta 3 (aVb3). According to our analysis, the integrin β3 expression is sustained in the case of adenomyosis, where no significant differences were detected in any phases of the menstrual cycle. While these results demonstrate a physiological expression pattern of this protein in adenomyosis, the other chain of the heterodimer is also necessary to maintain its activity and hence requires further investigation. 

To exert its action during the process of implantation, the aVb3 receptor depends on its ligand protein, namely osteopontin. Osteopontin is considered to be an indispensable mediator of maternal endometrium-embryo interactions, from promoting decidualization to ensuring successful communication between the uterus and the placenta [25]. A recent study found osteopontin to be among the most upregulated genes in the endometrium during the peri-implantation period, emphasizing its crucial role in establishing a successful pregnancy [26]. Moreover, progesterone has been found to directly stimulate osteopontin expression, so inadequate progesterone signaling may have a detrimental effect on osteopontin production and hamper embryo attachment [25,27]. In line with this theory, we observed a decreased osteopontin expression in adenomyosis, especially in the secretory-phase stroma, where it was significantly different from healthy endometrium in the same phase of the cycle. These results indicate that despite the physiological integrin β3 expression in adenomyosis, its action may be compromised by insufficient amounts of its ligand osteopontin, ultimately leading to impaired endometrial receptivity. 

Pinopodes are large cytoplasmic protrusions on the endometrial epithelium that develop during the WOI in response to progesterone [28]. They are considered a precondition for successful implantation, as they appear to be the exact site of embryo-endometrium interaction, where the embryo eventually attaches [24]. Two recent clinical trials have established the value of pinopodes for predicting reproductive outcomes in a clinical setting [29,30], but there are no explicit data on their involvement in adenomyosis. In this study, we did observe differences in pinopode formation between healthy endometrium and adenomyosis. In healthy endometrium, the epithelial surface was covered in fully formed and regularly shaped pinopodes interposed with ciliated cells, consistent with the physiological appearance of the endometrium during this period of the menstrual cycle. By contrast, a distinct image was observed in adenomyotic endometrium, with late-stage regressing pinopodes in some areas and complete absence in others. Although a larger sample size is essential to be able to draw solid conclusions on the role of pinopodes in adenomyosis-associated endometrial-factor infertility, our research provides the first evidence of inadequate and/or asynchronous development of pinopodes in affected subjects.

As previously described, both endometrial epithelial and stromal cells play distinct but complementary roles during implantation and subsequent progression of pregnancy [1]. While epithelium constitutes the site of initial apposition and adhesion, decidualized stroma provides the matrix that encapsulates the blastocyst and mediates trophoblast invasion [2,31]. We demonstrated increased rates of abnormalities within secretory-phase stroma from women affected by adenomyosis, with the glandular compartment from the same patients remaining relatively unchanged compared to healthy subjects. These findings have led us to hypothesize that inadequate stromal decidualization is the principal mechanism underlying poor IVF outcomes in adenomyosis patients. Although initial blastocyst implantation may be successful, failure of endometrial stroma to physiologically decidualize can inhibit trophoblast invasion and eventually cause spontaneous abortion. Consistent with this theory, most meta-analyses in the literature show comparable rates of implantation between women with and without uterine adenomyosis, with a significantly elevated risk of early miscarriage in the former group [5,6,7,8].

Our study has both strong points and limitations. On the plus side, we included a well-characterized and carefully selected patient population, taking into account different menstrual cycle phases to obtain a clearer picture of how the different proteins are expressed throughout. By immunohistochemistry, a technique allowing us to both visualize and quantify proteins, we could also assess the epithelial and stromal compartments separately and observe the differential behavior of these two important cell populations in adenomyotic endometrium. For the first time, we were able to determine the key role of stromal cells, showing irregularities in the expression of PR, LIF, HOXA10 and osteopontin.

One drawback of the study may be the high prevalence of leiomyomas in both healthy and adenomyosis subjects. Indeed, leiomyomas have been linked to subfertility, especially when located close to the endometrium. However, we found the rates of concurrent leiomyomas to be similar between the two groups, suggesting that the differential expression of investigated proteins cannot be blamed on this parameter (Table 1). Moreover, multiple regression analyses did not show leiomyomas to be a confounding factor in the expression of any of the proteins examined (Supplementary Table S2). Another limitation was probably that the study population had a higher mean age than their healthy counterparts (39.96 vs. 36.52). The uterus is generally considered an abiding reproductive organ (with respect to ovarian hormone secretion), but it has been postulated that endometrial receptivity may be partially influenced by advanced maternal age, although current data are contradictory [32,33]. We included only patients up to 45 years of age, as it has been previously demonstrated that endometrium itself may be less receptive above this threshold [34]. Furthermore, when considering age as an independent variable, it appeared to only affect PR expression without any other significant differences (Supplementary Table S2). We therefore believe that the altered expression of the different markers in adenomyosis cannot be attributed to the higher mean age in this group of patients. This difference in mean age may also account for increased mean parity in the adenomyosis group (Table 1). Indeed, in these patients, there was a multiple-year gap between childbirth and diagnosis of the condition, which likely hampered their fertility at a later point in time.

Overall, our pilot study provides new insights into endometrial-factor infertility in the case of adenomyosis. More extensive investigations into larger and more homogeneous populations are essential if we are to draw solid conclusions applicable to everyday clinical practice. It is crucial for future research to determine whether these receptivity markers can be used for predicting, diagnosing and improving poor reproductive outcomes in women suffering from adenomyosis.

## 5. Conclusions

Eutopic endometrium from adenomyosis patients is characterized by an abnormal expression of a number of receptivity markers, namely PR, LIF, HOXA10 and osteopontin. Our results show that most irregularities occur in the stromal compartment of adenomyosis patients, suggesting that stromal cells are pivotal to endometrial receptivity, as well as being more impacted by the presence of the disease. Multiple regression analysis confirmed that adenomyosis significantly affected the stromal HOXA10 expression when looking at age, menstrual cycle phase and concurrent presence of leiomyomas. We therefore hypothesize that this protein might be the most reliable indicator of endometrial-factor infertility in adenomyosis and could be taken into consideration when managing patients in a clinical context. All in all, our pilot study points to impaired endometrial decidualization being one of the mechanisms underlying poor reproductive outcomes in adenomyosis patients. Future research should focus on better interpreting these findings and implementing them in the clinical management of these patients. 

## Figures and Tables

**Table 1:** Clinicopathological characteristics of the patients involved in the study. Patients with adenomyosis were found to have higher mean age (* *p* = 0.0286) and parity (* *p* = 0.03) than healthy subjects. Rates of menorrhagia (* *p* = 0.0486) and dysmenorrhea (* *p* = 0.0481) were significantly higher in the adenomyosis group compared to the healthy group.

Parameter	Healthy	Adenomyosis	*p*-Value
Age (mean ± SD)	36.52 ± 6.125 Range = 21–43	39.96 ± 4.703 Range = 24–45	* 0.0286
Parity (mean ± SD)	1.2 ± 1.4 Range = 0–4	2 ± 1.3 Range = 0–4	* 0.03
Menstrual phase			
Proliferative	10	10	
Secretory	12	12	
Menstrual	4	4	
Menorrhagia (%)	37.5	65.38	* 0.0486
Dysmenorrhea (%)	16.67	42.31	* 0.0481
Leiomyoma (%)	45.83	38.46	0.5977

## Data Availability

Data will be shared on reasonable request to the corresponding author.

## Supplementary Materials

The following supporting information can be downloaded at: https://www.mdpi.com/article/10.3390/jcm13175016/s1, Figure S1: Representative pictures of positive control tissues; Table S1: Antibodies and parameters used for immunohistochemistry; Table S2: Multiple regression analysis using epithelial or stromal expression as the dependable variable.
