# Supplementary material for: Expression of Endometrial Receptivity Markers throughout the Menstrual Cycle in Women with and without Uterine Adenomyosis
Source: J Clin Med. 2024 Aug 24;13(17):5016. doi: 10.3390/jcm13175016 (PMC11395822; doi:10.3390/jcm13175016)
Supplement: Supplementary file 1 [file jcm-13-05016-s001.zip › jcm-3121391-supplementary.pdf]

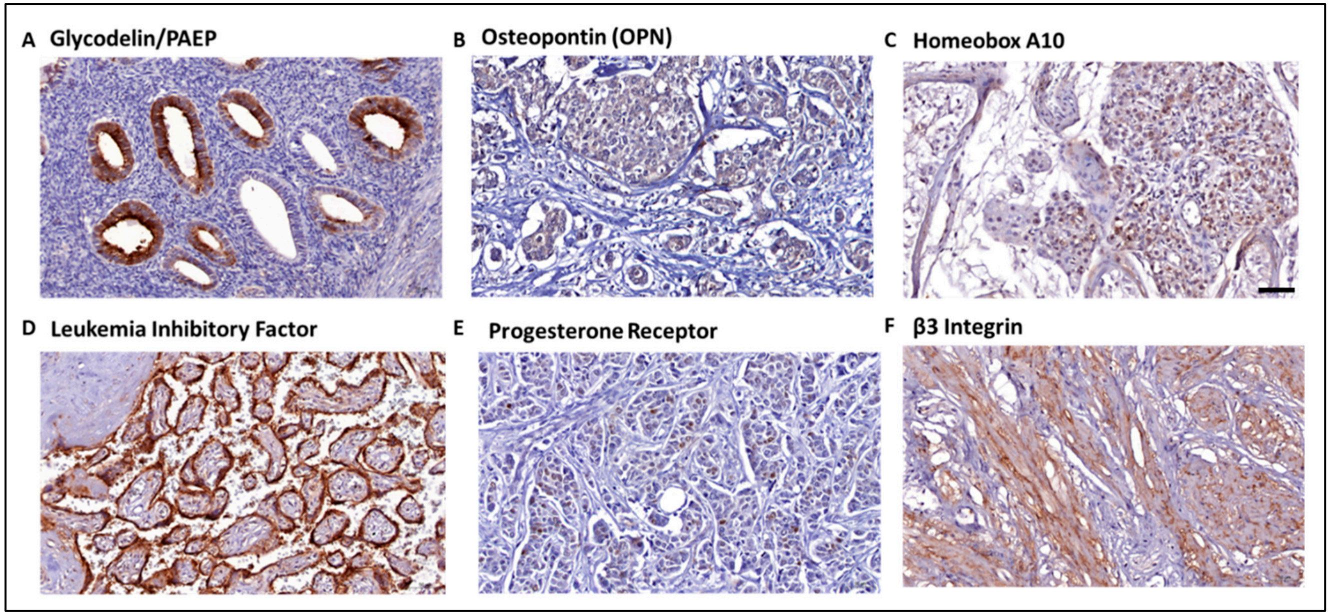

Supplementary figure S1. Representative pictures of positive control tissues used in immunohistochemistry. (**A**) Secretory endometrium for glycodelin. (**B**) Breast carcinoma for osteopontin. (**C**) Testis for HOXA10. (**D**) Placenta for LIF. (**E**) Breast carcinoma for PR. (**F**) Urinary bladder for β3 integrin.

Scale bar: 50 µm.

**Marker**	**EP blocking**	**Antigen retrieval**	**Primary antibody**	**Secondary antibody**	**Chromogen & counterstain**	**Positive/negative control**
Glycodelin (PAEP, PA5-54152, Thermofisher)	0.3% H_2O_2	0.01M sodium citrate buffer pH=6 with 20% Triton X-100 (75 min at 98°C)	Polyclonal Rabbit anti-PAEP; 1:1000 in TBS + 1% FBS, 0.1% BSA (4°C O/N)	Envision anti-rabbit, system HRP (60 min at RT; 1:1) #K4003, Dako	DAB & hematoxylin	Secretory endometrium/endometrium
Osteopontin (OPN, ab69498, Abcam)	0.3% H_2O_2	0.01M sodium citrate buffer pH=6 with 20% Triton X-100 (75 min at 98°C)	Monoclonal Mouse anti-OPN; 1:500 in Tris, 2.5% BSA, 1% Non-fat milk (4°C O/N)	Envision anti-mouse, system HRP, (60 min at RT; 1:2 in Tris, 2.5% BSA, 1% Non-fat milk) #K4001, Dako	DAB & hematoxylin	Breast cancer/endometrium
Homeobox A10 (HOXA10, BS-2502R, Thermofisher)	0.3% H_2O_2	0.01M sodium citrate buffer pH=6 with 20% Triton X-100 (75 min at 98°C)	Polyclonal Rabbit anti-HOXA10; 1:300 in Tris, 2.5% BSA, 1% Non-fat milk (4°C O/N)	Envision anti- rabbit, system HRP (60 min at RT; 1:1)	DAB & hematoxylin	Testis/endometrium
Leukemia inhibitory factor (LIF, PA5-79600, Thermofisher)	0.3% H_2O_2	0.01M sodium citrate buffer pH=6 with 20% Triton X-100 (75 min at 98°C)	Polyclonal Rabbit anti-LIF; 1:300 in TBS + 1% NGS, 0.1% BSA (4°C O/N)	Envision anti- rabbit, system HRP (60 min at RT; 1:1)	DAB & hematoxylin	Placenta/endometrium
Progesterone Receptor (PR, A0098, Dako)	3% H_2O_2	0.01M sodium citrate with 20% Triton X-100 (75 min at 98°C)	Polyclonal Rabbit anti-PR, 1:500 in TBS + 1% FBS + 0.1% BSA (4°C O/N)	Envision anti-rabbit, system HRP (60 min at RT; 1:1)	DAB & hematoxylin	Breast cancer/endometrium
β3 Integrin (ITG B3, ZRB1515, Merck)	0.3% H_2O_2	0.01M sodium citrate with 20% Triton X-100 (75 min at 98°C)	Monoclonal Rabbit IgG1 anti-ITGB3; 1:100 in TBS + 1% FBS, 0.1% BSA (4°C O/N)	Envision anti-rabbit, system HRP, (60 min at RT; 1:1)	DAB & hematoxylin	Urinary bladder/endometrium

Supplementary table S1. Antibodies and parameters used for immunohistochemistry.

EP: endogenous peroxidase; H_2O_2: hydrogen peroxide; TBS: tris-buffered saline; FBS: fetal bovine serum; BSA: bovine serum albumin; O/N: overnight; RT: room temperature; DAB: 3,3′-Diaminobenzidine.

Protein	Cell type	P-values				
		Age	Phase (S)	Phase (M)	Myoma	Adenomyosis
**PR**	Epithelium	0.4066	*0.0441	0.8849	0.6705	0.8029
	Stroma	*0.0241	0.3864	0.9919	0.8617	0.3932
**LIF**	Epithelium	0.9375	*0.0324	0.0681	0.329	0.4439
	Stroma	0.502	*0.0311	0.4667	0.6335	0.1717
**Glycodelin**	Epithelium	0.4510	****<0.0001	****<0.0001	0.1837	0.2609
**Osteopontin**	Epithelium	0.9126	**0.0094	0.0566	0.5341	0.2092
	Stromal	0.246	0.539	0.2899	0.7791	0.1139
**HOXA10**	Epithelium	0.5010	0.725	0.6928	0.6084	0.0746
	Stroma	0.3935	0.6593	0.751	0.222	**0.0029
**Integrin β3**	Epithelium	0.7453	0.2517	*0.0209	0.5792	0.1248
	Stromal	0.4470	0.5764	**0.0093	0.5369	0.4153

Supplementary Table S2. Multiple regression analysis using epithelial or stromal expression as the dependable variable. Advancing age was found to have a significant impact on stromal expression of PR (*p=0.0241). Secretory phase appeared to be the major factor determining expression of the different proteins, affecting stromal expression of PR (*p=0.0441), epithelial (*p=00324) and stromal (*0.0311) expression of LIF, rates of glycodelin-positive glands (****p<0.0001) and epithelial expression levels of osteopontin (**p=0.0094). Menstrual phase in term significantly affected expression of glycodelin (****p<0.0001) and epithelial (*p=0.00209) and stromal integrin β3 (**p=0.0093). The presence of myoma did not influence any of the proteins examined. Last, the presence of adenomyosis was identified as an independent variable significantly affecting stromal expression of HOXA10 (**p=0.0029).

PR: progesterone receptor; LIF: leukemia inhibitory factor; HOXA10: homeobox A10.
